# Cancer Pain Management: Opioid Analgesics, Part 2

**Published:** 2017-09-01

**Authors:** Rita J. Wickham

**Affiliations:** Rush University College of Nursing (Adjunct Faculty), Chicago, Illinois

## Abstract

Opioid analgesics are the cornerstone of moderate to severe cancer pain management, and do not have ceiling doses unless unmanageable adverse effects occur. Oral, short-acting pure μ agonists such as morphine are most frequently used, but other agents and administration formulations allow finding the right opioid and dose for most patients. In addition, clinicians must understand the metabolism, pharmacokinetics, and elimination of particular drugs to individualize opioid selection, select initial doses, and appropriately escalate doses to satisfactory pain relief or uncontrollable toxicity. Anticipation and proactive management of possible adverse effects, particularly constipation, confusion or delirium, opioid-specific adverse effects, and opioid abuse, are also integral to primary and secondary prophylaxis and management.

Opioids—mu (μ) agonists and buprenorphine—are essential for managing moderate to severe cancer pain. They have no analgesic ceiling, and doses can usually be escalated to pain relief without unmanageable side effects. Key strategies are to individualize opioid doses and schedules, weigh benefits and burdens, and anticipate possible adverse effects with an eye toward prophylaxis and effective, timely management. 

The first part of this series on cancer pain management addressed nonopioid analgesics. It can be found in the July/August 2017 issue of *JADPRO*, or on advancedpractitioner.com

## OPIOID METABOLISM AND PHARMACOLOGY

Opioids activate the endogenous pain-modulating system of opioid peptides (e.g., enkephalins, endorphins, and dynorphins) and spinal cord and brain receptors (μ, kappa [κ], and delta [δ]) to alter pain perception (Branford, Droney, & Ross, 2012; Lam, Pirrello, & Ma, 2016; Pasternak, 2014). The μ agonists also indirectly modify descending spinal cord inhibitory pathways (Pathan & Williams, 2012). 

Metabolism biotransforms some drugs to intermediate metabolites, which are potentially more potent than a parent drug, or to inactive (and possibly toxic) metabolites, which all must be detoxified and excreted (Table 1). Some unchanged opioids act at receptors, but most must be metabolized to active forms (Mercadante, 2015; Smith, 2011). During phase 1 metabolism, cytochrome P450 (CYP) enzymes modify opioids, largely by hydroxylation or oxidation. Almost 50% of drugs are metabolized by CYP3A4 and have the highest risk for drug-drug interactions, and 25% are metabolized by CYP2D6 enzymes and have an intermediate risk for drug-drug interactions. This risk is minimal with drugs that undergo phase 2 metabolism, most commonly by uridine diphosphate glucuronosyltransferase (UGT) 2B7 glucuronidation, which conjugates drugs or metabolites to hydrophilic, excretable products. Age, disease, genetic factors, and route of administration may also alter metabolism, distribution, half-life, and excretion (Bosilkovska, Walder, Besson, Daali, & Desmeules, 2012; Brant, 2010).

**Table 1:**
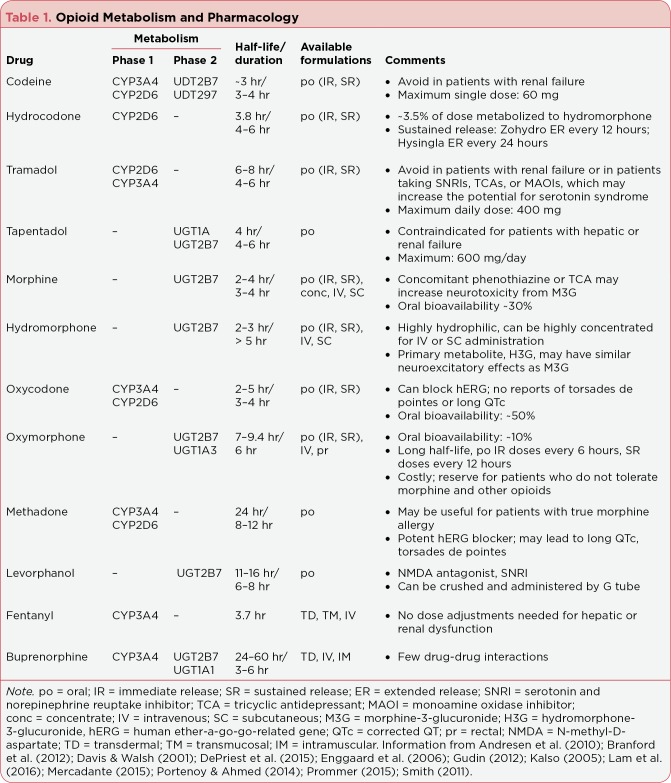
Opioid Metabolism and Pharmacology

All CYP genes are highly polymorphic, but CYP2D6 is of particular interest because polymorphisms directly or indirectly affect the pharmacokinetics of half of all drugs (De Gregori et al., 2016). More than 100 CYP2D6 allelic variants (Ingelman-Sundberg, n.d.) fall into 4 phenotypic groups. Most people are extensive metabolizers (EMs) with two functional alleles, or intermediate metabolizers (IMs) with one functional and one nonfunctional allele. Fewer are poor metabolizers (PMs), who have defective metabolism secondary to inactivating mutations or deletions of both alleles, or ultrarapid metabolizers (UMs) who express multiple copies of greater-than-normal function enzymes (Gudin, Fudin, & Nalamachu, 2012). 

**Figure 1:**
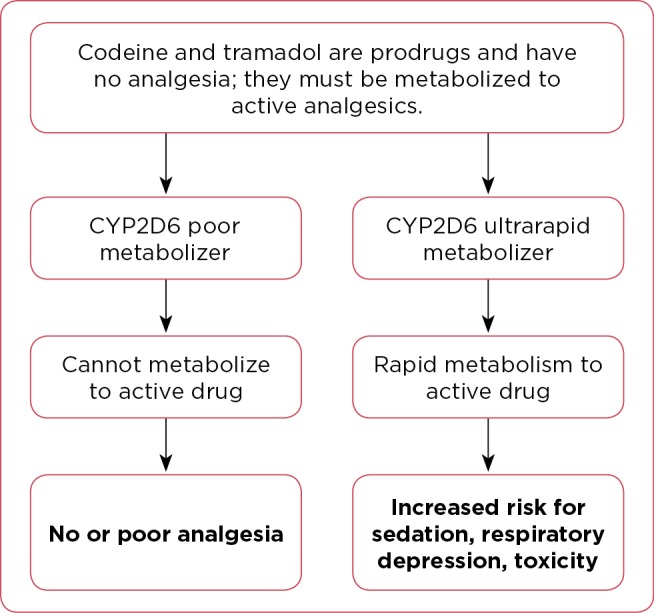
Impact of CYP2D6 gene inheritance on prodrug metabolism. Information from Crews et al. (2012); Kirchheiner et al. (2007); Leppert (2011); PharmGKB (2017); Smith (2009); Tassinari et al. (2011).

The clinical significance for PMs and UMs depends on whether the parent drug or metabolite is analgesic (Figure 1). Codeine and tramadol are prodrugs that CYP2D6 metabolizes to active analgesics; PMs attain no or poor relief, and UMs have a high risk for toxicity (Enggaard et al., 2006; Kirchheiner et al., 2007; Leppert, 2011; Mercadante, 2015). Polymorphisms in other genes, such as UGT2B7, genes for μ receptors, and opioid transport proteins may also affect analgesia (Branford et al., 2012; Nalamachu, 2012; Tremblay & Hamet, 2010; Trescot & Faynboym, 2014). 

## WHICH OPIOID ANALGESIC TO PRESCRIBE?

Some clinicians start opioid-naive patients with moderate pain on a "weak" oral (po) opioid: codeine, hydrocodone, or tramadol with or without acetaminophen (APAP). Others prescribe a small dose of a "strong" opioid: po morphine at 5 to 15 mg or the equivalent every 4 hours, eliminating the need to switch opioids if the pain worsens (Caraceni et al., 2012; Klepstad, Kaasa, & Borchgrevink, 2010; Marinangeli et al., 2004). Formulations include po regular (IR) and sustained release (SR), rectal (pr), intravenous (IV), subcutaneous (SC), intramuscular (IM), transmucosal (TM), transdermal (TD), or intraspinal (IS).

**Codeine (po [IR])**

Codeine, a weak μ and δ agonist, may be less effective than a nonsteroidal anti-inflammatory drug (NSAID), and combinations with APAP are slightly better than APAP alone (Prescrire Editorial Staff, 2015). CYP3A4 and UDT2B7 enzymes metabolize 80% of codeine to almost inactive metabolites, and CYP2D6 metabolizes 5% to 10% of this prodrug to morphine (Crews et al., 2012). Although 77% to 92% of people are CYP2D6 EMs or IMs, PMs cannot metabolize codeine to morphine and derive no analgesia. Ultrarapid metabolizers rapidly and extensively convert codeine and may experience sedation, confusion, and shallow or slow respirations (PharmGKB, 2017). Patients with renal failure should not take codeine (Kirchheiner et al., 2007; Smith, 2009). 

**Tramadol (po [IR, SR])**

Tramadol is a weak μ agonist and a serotonin and norepinephrine reuptake inhibitor (SNRI; Fukshansky, Are, & Burton, 2005; Prommer, 2015). Tramadol with or without APAP is not superior to ibuprofen, hydrocodone, codeine, or low-dose morphine (Prescrire Editorial Staff, 2015; Tassinari et al., 2011). It is a prodrug metabolized by CYP2D6; PMs achieve poor analgesia, and UMs experience toxicities. Respiratory depression is rare but most common in UMs or persons with renal dysfunction (Enggaard et al., 2006; Leppert, 2011; Orliaguet et al., 2014; Stamer, Stuber, Muders, & Musshoff, 2008). 

CYP2D6 poor metabolizers and those with deficient serotonin (5-HT) uptake who take tramadol may be at risk for serotonin syndrome (SS), which begins within 24 hours of starting or increasing a 5-HT reuptake or CYP2D6 inhibitor (Beakley, Kaye, & Kaye, 2015; Houlihan, 2004; Leppert, 2009). Table 2 outlines the manifestations of mild to life-threatening SS, which occur because serotonin accumulates at 5-HT receptors in the central nervous system (Werneke, Jamshidi, Taylor, & Ott, 2016). Diagnosis is based on clinical findings and is managed by stopping offending drugs and supportive measures for agitation, hyperthermia, and autonomic dysfunction. 

**Table 2:**
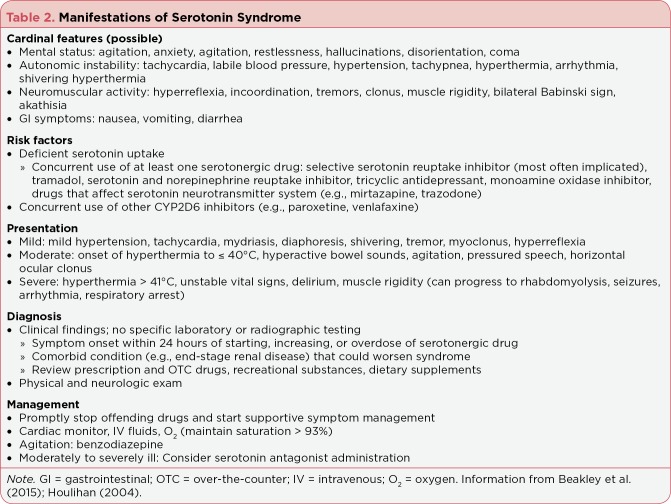
Manifestations of Serotonin Syndrome

Seizures after tramadol ingestion have been reported, usually after intentional or accidental overdose or abuse. Most reports originate in Europe or the Middle East, where tramadol abuse is more common. For instance, tramadol was implicated in 4.8% of seizures reported to a Swiss poison center (Reichert et al., 2014). 

**Tapentadol (po [IR, SR])**

Tapentadol is a high-affinity μ, δ, and κ receptor agonist as well as a strong SNRI. It undergoes phase 2 glucuronidation and minor phase 1 CYP2D6 metabolism to inactive metabolites excreted in urine (Prommer, 2015). Tapentadol studies were often limited by high dropout rates, missing data, and nonsuperiority to other opioids (Veal & Peterson, 2015; Wiffen, Derry, Naessens, & Bell, 2015). A comparative US study confirmed tapentadol was associated with greater risks for hallucinations or delusions, respiratory depression, and coma, whereas large tramadol doses were associated with a greater risk for seizures (Tsutaoka, Ho, Fung, & Kearney, 2015). Rare cases of SS have been reported with tapentadol, which is contraindicated for patients with hepatic or renal failure (US Food and Drug Administration [FDA], 2016); Guay, 2009). 

**Hydrocodone (po [IR, SR])**

Hydrocodone, classified as a "weak" opioid, is similarly effective (mg per mg) to morphine (Fukshansky et al., 2005). Hydrocodone is metabolized by CYP2D6, but only the parent drug and one metabolite (hydromorphone) are analgesic, so CYP2D6 status likely has no major clinical significance (Barakat, Atayee, Best, & Pesce, 2012; Smith, 2011). Prescriptions for hydrocodone formulated with ibuprofen or APAP (300 or 325 mg) must be exact (e.g., hydrocodone 10/325, # 120 [one hundred twenty]). There are two SR hydrocodone products; brand names must be prescribed because doses and schedules differ. 

**Morphine (po [IR, SR], IV, SC)**

Morphine is a μ agonist with minor κ and δ agonist effects at high doses (Bosilkovska et al., 2012; Pathan & Williams, 2012). Most morphine is metabolized by UGT2B7 (phase 2): 10% to morphine-6-glucuronide (M6G) and 50% to morphine-3-glucuronide (M3G; DePriest, Puet, Holt, Roberts, & Cone, 2015; Fukshansky et al., 2005). M6G is a longer-acting, more potent μ agonist than morphine, but M3G cannot bind at opioid receptors and is not analgesic (Mercadante, 2015).

Liver impairment does not impact dosing, but there is an inverse relationship between renal function and excretion. Morphine must be titrated cautiously when creatinine clearance is < 30 mL/minute to avoid M3G-related neurotoxic effects, which are more common with morphine than oxycodone to TD fentanyl or buprenorphine (Corli et al., 2016; Donnelly, Davis, Walsh, & Naughton, 2002; Portenoy & Ahmed, 2014; Smith, 2011). 

**Oxycodone (po [IR, SR])**

Oxycodone has lower binding affinity for μ receptors than does morphine (Olkkola, Kontinen, Saari, & Kalso, 2013). Phase 1 metabolism results in noroxycodone, a weak agonist, and oxymorphone, which are excreted in the urine (Kalso, 2005). Oxycodone has greater bioavailability and is less likely than morphine to cause hallucinations, nausea, sleepiness, and pruritus (Caraceni et al., 2012; King, Reid, Forbes, & Hanks, 2011). 

**Hydromorphone (po [IR, SR], IV, SC)**

Hydromorphone, a μ and δ agonist, is structurally similar to morphine but more lipid soluble (Pigni, Brunelli, & Caraceni, 2010; Wirz, Wartenberg, & Nadstawek, 2008). It is metabolized to minor metabolites and glucuronidated to hydromorphone-3-glucuronide (H3G), which has no analgesia but may be neurotoxic (Murray & Hagen, 2005; Smith, 2011; Vallejo, Barkin, & Wang, 2011). Hydromorphone-3-glucuronide may accumulate with renal insufficiency and increase toxicity. Parenteral hydromorphone can be highly concentrated (≥ 100 mg/mL) and is useful for patients who require large-dose, small-volume continuous IV (CIV) or SC ambulatory infusions (Kumar & Lin, 2007). 

**Oxymorphone (po [IR, SR], pr)**

Oxymorphone is a complex, potent, highly selective μ receptor agonist. Immediate-release po doses are more useful for acute, moderate pain, whereas SR doses are preferable for severe, persistent cancer pain (Smith, 2009), but it is rarely used because of its high cost. Oxymorphone has higher μ-binding affinity and a longer half-life than morphine (Mayyas, Fayers, Kaasa, & Dale, 2010; Prommer, 2006). With regular dosing, steady state occurs in 3 to 4 days. Oral oxymorphone is more lipophilic than oxycodone and morphine, and doses are rapidly absorbed from the gut to undergo extensive first-pass metabolism. Phase 2 hepatic and renal UGT2B7 glucuronidation results in major metabolites, 6-hydroxy-oxymorphone (6-OH-OXM), which is analgesic, and oxymorphone-3-glucuronide (OXM-3G), which has undefined properties (Prommer, 2006). Oxymorphone has no clinically significant CYP interactions or common drug-drug interactions (Smith, 2009), and up to 2% of the parent drug is excreted in the urine. Oxymorphone accumulates in renal failure (Gudin, 2012). 

**Levorphanol (po)**

Levorphanol, a μ, κ, and δ receptor agonist, is also an SNRI and N-methyl-D-aspartate (NMDA) antagonist (Pham, Fudin, & Raffa, 2015; Prommer, 2014). N-methyl-D-aspartate receptors are activated in chronic pain; antagonists may alleviate pain unresponsive to opioids and reverse tolerance. Levorphanol is more potent than methadone and has few drug interactions but is expensive and rarely used since SR opioids became available (Gudin et al., 2016; McNulty, 2007). Its long half-life (11–16 hours) means baseline doses should not be increased before steady state is reached (2–3 days). Table 3 shows the equianalgesic ratio of levorphanol (and methadone) to morphine varies by dose (Loitman, 2011). Phase 2 metabolism results in inactive metabolites excreted in urine. Hepatic or renal insufficiency may result in neurotoxic metabolite accumulation, dictating extended dosing intervals (Pham et al., 2015). 

**Table 3:**
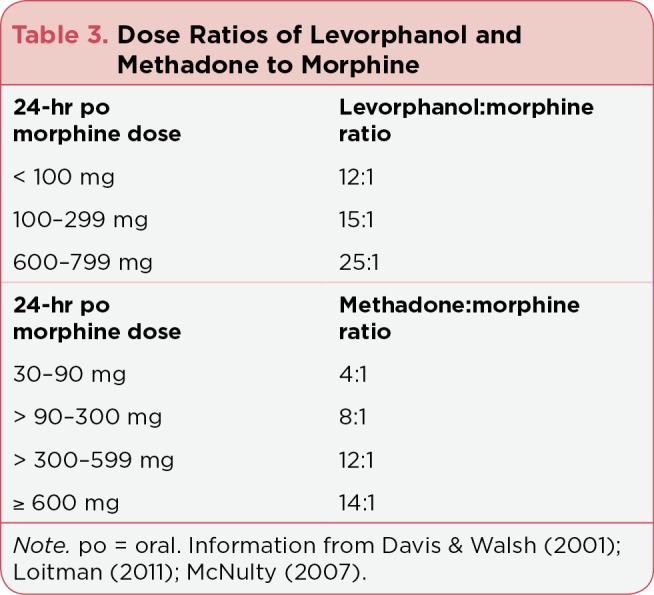
Dose Ratios of Levorphanol and Methadone to Morphine

**Methadone (po)**

Methadone is a low-cost potent, lipophilic μ agonist, SNRI, and NMDA antagonist that has high oral and rectal absorption, long duration of action, and no neurotoxic metabolites (Davis & Walsh, 2001; Mercadante, 2015; Prommer, 2015; Smith, 2011). Highly variable pharmacokinetics complicate the use of methadone: the half-life averages 24 hours but ranges from < 15 to > 130 hours (Portenoy & Ahmed, 2014). Methadone is primarily metabolized by CYP3A4 and CYP2B6; as a CYP2D6 inhibitor, it may block metabolism and increase accumulation (Trescot & Faynboym, 2014). Usual dosing intervals are 8 or 12 hours, along with short-acting rescue (as needed [prn]) opioid doses (Ripamonti, Santini, Maranzano, Berti, & Roila, 2012). Collaborating with other colleagues to change a patient from another opioid to methadone enhances knowledge, clinical skills, and patient safety (Fine & Portenoy, 2009). 

**Fentanyl (TD, IV)**

Fentanyl is a rapid-onset, lipophilic μ agonist 50 to 100 times more potent than morphine (Pathan & Williams, 2012). Continuous or repeated dosing (TD or IV administration) increases its half-life redistribution and accumulation to muscle and fat (DePriest et al., 2015). Concomitant administration with strong CYP3A4 inhibitors (e.g., clarithromycin, ketoconazole, itraconazole, and grapefruit juice) may affect its metabolism (Fukshansky et al., 2005). Although more than 90% of fentanyl and its metabolites are excreted in the urine, it is safe for patients with renal dysfunction. 

**Buprenorphine (TD, IV, IM)**

Buprenorphine is a lipophilic μ receptor agonist opioid with no analgesic ceiling, a partial agonist (ceiling) for respiratory depression, and a κ receptor antagonist (Clemens, Faust, Jaspers, & Mikus, 2013; Pergolizzi et al., 2010; Virk, Arttamangkul, Birdsong, & Williams, 2009). It is 25 to 50 times more potent than morphine, and high affinity binding/slow dissociation from μ receptors allows buprenorphine to displace other agonists from the μ receptor, which may overcome opioid dependence and decrease tolerance (Prommer, 2015). It has antihyperalgesic effects, possible efficacy for neuropathic pain, low rates of constipation (1%–5%), and minimal endocrine effects. Buprenorphine undergoes phase 1 metabolism to norbuprenorphine and secondary phase 2 glucuronidation. It is safe for patients with mild to moderate liver dysfunction, but severe liver disease may inhibit its metabolism (Khanna & Pillarisetti, 2015). Only 10% to 30% is excreted in the urine, so buprenorphine is safe for patients with renal impairment, even for those undergoing dialysis (Davis, 2012).

## ADVERSE EFFECTS OF OPIOIDS

An effective opioid and dose for one patient may cause dose-limiting adverse effects for another patient. This highlights the importance of patient teaching, follow-up assessment, and proactive management (summarized in Table 4). 

**Table 4:**
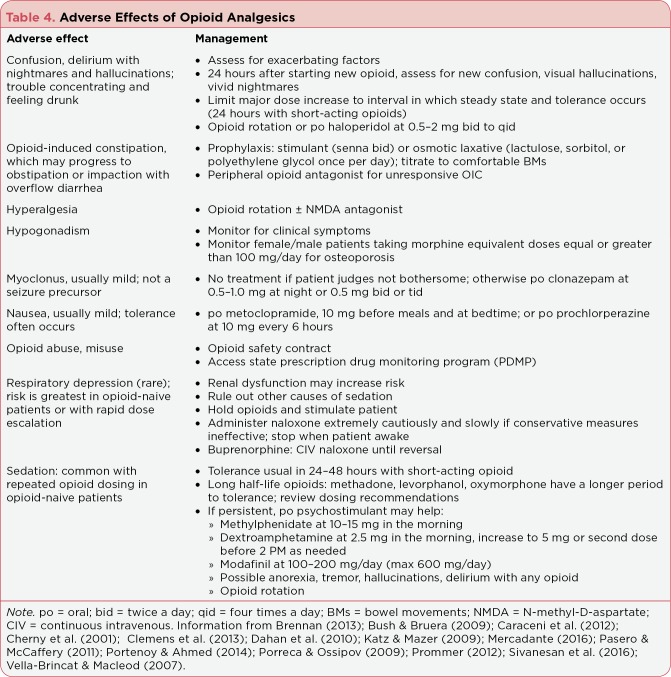
Adverse Effects of Opioid Analgesics

**Prolonged Cardiac Repolarization**

Prolonged QT interval (QTc) is a rare, potentially serious adverse effect of methadone and other drugs that can block human ether-a-go-go gene (hERG)-related potassium ion channels and alter cardiac repolarization (Krantz, Martin, Stimmel, Mehta, & Haigney, 2009). The normal QTc—measured from the start of the QRS to the end of the T wave—is < 450 milliseconds (ms) in women and < 430 ms in men; prolonged QTc is > 470 ms and > 450 ms, respectively. Methadone-induced long QTc (> 500 ms) is dose-related, asymptomatic, and usually resolves spontaneously. It rarely progresses to palpitations, syncope, seizures, torsades de pointes (torsades; a ventricular tachycardia), or sudden cardiac death (Isbister, 2015; Portenoy & Ahmed, 2014; Stringer, Welsh, & Tommasello, 2009).

The incidence of long QTc is undefined. One prospective study identified it in 4.6% of 173 individuals receiving maintenance methadone ≥ 120 mg/day (and none taking buprenorphine); mortality was merely 0.06 per 100 patient-years (Anchersen, Clausen, Gossop, Hansteen & Waal, 2009). Consensus-based guidelines (Table 5) address concerns and aid prescribing methadone (Chou et al., 2014). 

**Table 5:**
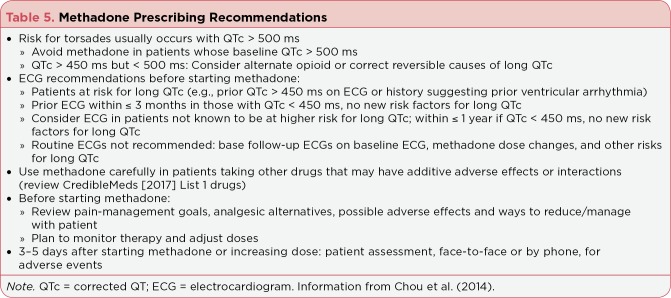
Methadone Prescribing Recommendations

Drug-induced long QTc is twice as common in blacks as in whites or Hispanics (Manini, Stimmel, & Vlahov, 2014). Other strongly related risks include demographic factors (age > 65 years, female gender, and smoking), cardiovascular problems (ischemic cardiomyopathy, hypertension, and arrhythmia), electrolyte disturbances (hypokalemia and hypocalcemia), and cardiovascular and other list 1 drugs (CredibleMeds, 2017; Vandael, Vandenberk, Vandenberghe, Willems, & Foulon, 2017). CredibleMeds categorizes drugs in three lists: (1) known risk of torsades (e.g., antiarrhythmics, antibiotics, antidepressants, antipsychotics, anticancer agents, methadone, and ondansetron); (2) possible risk; or (3) conditional risk. 

Vandael and colleagues (2017) found no relationship between prolonged QTc or torsades and lists 2 or 3 drugs. Buprenorphine is a CredibleMed list 2 drug (possible risk), and others have characterized QTc increases as modest (< 10 ms) and almost irrelevant (Darpo et al., 2016; Keller, Ponte, & Di Girolamo, 2010). Package inserts list a concern about hERG-related torsades (Purdue Pharma, 2017), but only 10 cases of torsades in people receiving buprenorphine (along with other drugs associated with torsades) were reported to the FDA over 19 years (Kao, Haigney, Mehler, & Krantz, 2015). Furthermore, the lowest European TD dose exceeds the highest recommended US dose (35 vs. 20 mcg/hour) with no more reports of long QTc or torsades. There are rare reports of hERG-associated prolonged QTc (but not torsades) with other opioids, including oxycodone overdose and tramadol in patients with renal failure (Berling, Whyte, & Isbister, 2013; Keller et al., 2016). 

**Constipation**

Opioid-induced constipation (OIC) occurs because of binding to gastrointestinal (GI) tract opioid receptors and slows motility with fewer, harder bowel movements (BMs; Brock et al., 2012). Exacerbating factors include disease or metabolic problems (e.g., hypercalcemia or diabetes), other drugs (e.g., anticholinergic agents or iron), low physical activity, and inadequate fluid intake (Cherny et al., 2001). Nondrug measures, fiber, or stool softeners cannot prevent or treat OIC, and prophylaxis with a stimulant or osmotic laxative, titrated to comfortable BMs, is crucial (Clemens et al., 2013; Wickham, 2017). Gastrointestinal opioid receptor antagonists (e.g., methylnaltrexone) are indicated only if other laxatives are ineffective and OIC is the only cause of constipation (Rauck, 2013). 

**Drowsiness or Sedation**

Initial opioid-related sedation *may* be beneficial if it allows restful sleep, but safety is a concern until tolerance develops, usually in 3 to 7 days with short-acting opioids. Psychostimulants may counteract persistent sedation and cognitive impairment and enhance analgesia, but evidence is limited (Caraceni et al., 2012; Mercadante, 2016; Portenoy & Ahmed, 2014; Webster, Andrews, & Stoddard, 2003). Methylphenidate, modafinil, or dextroamphetamine are commonly recommended and have narrow therapeutic indices. They may induce anorexia, tremor, hallucinations, delirium, or psychosis (Cherny et al., 2001). Methylphenidate also has a rapid-onset antidepressant effect in terminally ill individuals (Prommer, 2012). 

**Opioid-Induced Delirium**

Opioid-induced delirium (OID) is most common in patients starting tramadol or morphine, but it occurs with other opioids (Corli et al., 2016; Sivanesan, Gitlin, & Candiotti, 2016). About 10% to 15% of patients experience OID, possibly with visual hallucinations, altered mood, and drowsiness (Vella-Brincat & Macleod, 2007; Wang, Sands, Vaurio, Mullen, & Leung, 2007). Patients may experience other features of delirium, such as disorientation, impaired memory, and nightmares or vivid dreams. Delirium is often multicausal and worsened by other drugs (benzodiazepines, antidepressants, steroids, and anticholinergic agents), sepsis, organ failure, dehydration, or brain metastases (Chowdhury & Board, 2009). It is easy to miss mild confusion, and patients who feel "crazy" and upset typically do not volunteer such information. However, 74% remember delirium after recovery (Bush & Bruera, 2009). 

Baseline and subsequent mental status assessment is essential. The Confusion Assessment Method (CAM) is a brief, free to use, sensitive clinical tool to aid rapid identification (Wei, Fearing, Sternberg, & Inouye, 2008). Alternately, the day after starting a new opioid, *routinely* ask patients about feeling confused or having vivid dreams, nightmares, or visual hallucinations (Sivanesan et al., 2016). Switching to a different opioid is a practical and effective intervention for more than 80% of patients (Mercadante, 2016). Other clinicians start haloperidol at 2 mg/day and titrate against delirium or to adverse effects (Vella-Brincat & Macleod, 2007).

**Nausea and Vomiting**

Nausea (N) or vomiting (V) can occur with new opioids secondary to decreased GI motility, chemoreceptor trigger zone stimulation, or vestibular apparatus activation and often resolves in 1 or 2 weeks (Cherny et al., 2001; Porreca & Ossipov, 2009). A dopamine antagonist (prochlorperazine or haloperidol) or metoclopramide is a first-line antiemetic, and serotonin antagonists (e.g., ondansetron) are second-line options. Opioid rotation is appropriate for intractable N and V (Caraceni et al., 2012). 

**Hypogonadism**

Up to 90% of those taking opioids for chronic pain develop dose-related hypogonadism. Opioids bind to receptors in the hypothalamus, pituitary, and testes, leading to downstream decreases in sex hormones, adrenal androgen, cortisol, and testosterone (Brennan, 2013; Buss & Leppert, 2014). Net effects are amenorrhea in women or erectile dysfunction in men, decreased libido, vasomotor instability, infertility, muscle loss and corresponding visceral fat increase, and fatigue (De Maddalena, Bellini, Berra, Meriggiola, & Aloisi, 2012; Katz & Mazer, 2009).

Opioids also bind to osteoclast receptors, accelerating bone loss and reducing bone formation. Although more common in women, almost half of all men with chronic opioid intake develop osteoporosis (Brennan, 2013), so bone density should be monitored in patients taking doses equivalent to morphine ≥ 100 mg/day, especially in patients with other risks for osteoporosis and fractures. If the benefits outweigh the risks, testosterone replacement may help men whose free and bound serum testosterone is < 300 ng/dL (Portenoy & Ahmed, 2014). Clinical findings may identify women who could benefit from androgen replacement with over-the-counter dehydroepiandrosterone (DHEA; Colameco & Coren, 2009).

**Respiratory Depression**

Dahan, Aarts, and Smith (2010) reviewed multiple meta-analyses and clinical reports of patients receiving parenteral or epidural opioids for cancer-related, traumatic or postsurgical pain. They concluded that up to 0.5% of such patients experience opioid-induced respiratory depression (OIRD). Opioid-naive patients with acute pain are at the greatest risk in the first 24 hours after starting short half-life opioids, which can continue until steady state occurs, and risk largely resolves within 1 week (Pasero & McCaffery, 2011). Pain is usually an "antidote" for OIRD, but obese or elderly patients who snore (indicator of some airway obstruction) are at greater risk (Donnelly et al., 2002). Unexpected sedation in opioid-tolerant patients warrants exploration of other possible causes (e.g., sepsis, benzodiazepines, or hypercalcemia). 

Profound sedation precedes and accompanies OIRD; a sleeping, easily arousable patient who is not cyanotic does not have OIRD, whereas a sedated person with a low respiratory rate or shallow respirations and pinpoint pupils may (Pasero & McCaffery, 2011). Management begins by holding opioids and continuously stimulating the patient. If wakefulness does not increase, naloxone should be administered with *extreme caution* because of the risks for dangerous and life-threating adverse effects, especially in hypovolemic or hypotensive patients (Donnelly et al., 2002). Naloxone is a competitive μ receptor antagonist that induces dose-related agonist dissociation within 1 minute. Rapid IV doses cause massive catecholamine release with associated pulmonary edema, cardiac arrhythmia, hypertension, seizures, and even cardiac arrest—and immediate return of pain (Dahan et al., 2010). Each 0.4-mg ampule is admixed with saline to a volume of 10 mL; 0.5 to 1.0 mL is administered over 1 to 2 minutes while the patient’s cardiorespiratory status, level of consciousness, and pain are monitored.

Management of OIRD with buprenorphine—which is even rarer—is different because of its strong affinity/slow dissociation from μ receptors. The naloxone-buprenorphine dose response is bell-shaped: naloxone at 0.5 mg has little effect on OIRD but at 2 mg fully reverses OIRD within 40 to 60 minutes; however, naloxone at > 5 mg decreases the respiratory antagonist effect. A bolus of IV naloxone, 2 or 3 mg slowly administered, should be followed by CIV naloxone at 4 mg/hour given until symptoms completely reverse (Dahan et al., 2010; Pergolizzi et al., 2010). Opioid-induced respiratory depression from higher buprenorphine doses requires longer infusions. 

**Hyperalgesia**

Opioid-induced hyperalgesia (OIH)—poorly defined pain spreading beyond the initial site—occurs rarely in patients taking high opioid doses, especially with rapid dose escalation (Youssef, Pater, & Shehata, 2015). Opioid-induced hyperalgesia may involve spinal cord processes including activation of NMDA receptors, dynorphin activation of κ and NMDA receptors, descending pathway facilitation and sensitization of neuronal on cells that modulate transmission of painful stimuli, and decreased reuptake of nociceptive neurotransmitters. Management may be difficult and time-consuming, usually starting by rotation to another opioid and possibly adding an NMDA antagonist (e.g., IV ketamine, methadone, or buprenorphine) trial (Yi & Pryzbylkowski, 2015).

**Prescription Opioid Abuse**

Strategies to decrease opioid abuse include opioid safety contracts and prescription drug monitoring programs (PDMPs). An opioid safety contract might articulate how and when the patient will take opioid analgesic(s), mention keeping opioids in a lockbox, include an agreement to get prescriptions from one provider, and perhaps list recreational substances (e.g., marijuana) that will or will not be allowed (Barclay, Owens, & Blackhall, 2014). Related strategies include dispensing smaller opioid amounts (1 week supply); pill counts; and involving social workers, psychiatrists, and other professionals in substance abuse, pain medicine, or palliative care (Portenoy & Ahmed, 2014). High-risk patients might be monitored with random urine drug tests (UDTs) for illicit drugs, opioids not prescribed, and absence of prescribed drugs. Disadvantages are possible, such as inaccurately negative UDTs in rapid metabolizers taking prescribed opioids, an inability to determine drug serum concentration, and urine pH (Nafziger & Bertino, 2009; Trescot & Faynboym, 2014). 

A vital resource for prescribers is their state’s PDMPs, which are fully or partially operational in the District of Columbia and in all states except Missouri. Prescription drug monitoring programs are online databases of controlled substances and other drugs with misuse or abuse potential (National Association of Boards of Pharmacy, 2017). Within 3 days of dispensing a defined medication, a pharmacist enters basic patient and drug information into the PDMP database, which is accessible to authorized health-care practitioners or designated assistants, pharmacists, regulatory boards, and law enforcement agencies. Clinicians can readily review patients’ prescription drug histories before writing a new or renewed opioid analgesic prescription, and those near a state border can also access a nearby state PDMP. 

## EFFECTS OF ROUTE OF ADMINISTRATION

**Figure 2:**
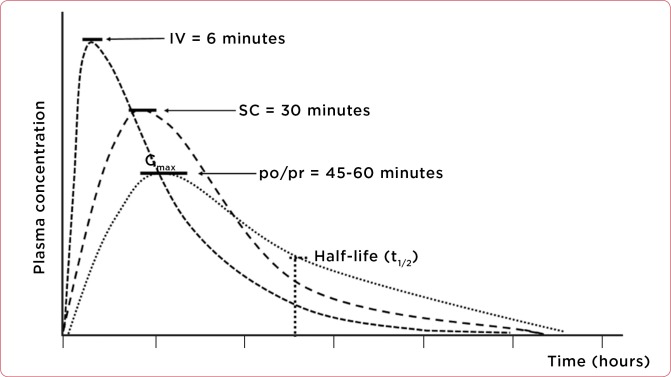
Opioid pharmacokinetic peak serum levels by route of administration. They are representative curves (and durations of action), and actual curves would vary somewhat by drug lipophilicity. However, peak serum levels are highest and occur soonest with IV administration, and lower and later with SC, and then immediate-release po or pr administration. Cmax represents the highest drug serum level and is followed by a peak analgesic effect as the opioid crosses the blood-brain barrier and reaches CNS opioid receptors. Systemic circulation, particularly through the liver and kidneys, means continued metabolism and excretion of drug and/or metabolites. IV = intravenous; SC = subcutaneous; po = oral; pr = rectal; CNS = central nervous system. Information from Collins et al. (1998); Hoskin et al. (1989); Kalso (2005); Pöyhiä et al. (1992); Smith (2009); Stuart-Harris et al. (2000).

Oral and pr opioids are directly absorbed from the GI tract into the hepatic circulation, undergo extensive first-pass metabolism, and have lower bioavailability than drugs given by other routes. Hydrophilic opioids (morphine, oxycodone, and hydromorphone) absorb slowly across the GI mucosa, cell membranes, and the blood-brain barrier, whereas lipophilic drugs (fentanyl, methadone, and buprenorphine) diffuse more rapidly. The route of administration also influences onset, peak level (C_max_), and duration of analgesia (Hoskin et al., 1989). Drugs distribute to serum, organs (e.g., lungs, kidneys, liver, and skeletal muscle), proteins, and fat. As Figure 2 shows, peak serum levels (C_max_) occur approximately 45 to 80 minutes after po or pr, 20 to 40 minutes after SC, and 6 to 20 minutes after IV opioid doses (Collins, Faura, Moore, & McQuay, 1998; Pöyhiä, Seppala, Olkkola, & Kalso, 1992; Stuart-Harris, Joel, McDonald, Currow, & Slevin, 2000). If pain is not adequately relieved within these times, prn rescue doses can be given every 2 hours for po doses, 45 minutes for SC doses, and 20 minutes for IV doses (Prommer, 2015).

Oral opioids are easiest to administer, whereas pr dosing is limited to short-term use in patients without po intake (e.g., dying at home). Intravenous dosing is advantageous for patients who require rapid dose escalation, have GI obstruction or unrelenting nausea, or when large po doses are impractical (Prommer, 2015). The bioavailability of SC opioids is about 78% of IV doses, but IM doses are variably absorbed, painful, and generally discouraged (Kumar & Lin, 2007). Persons taking adequate doses at appropriate intervals around-the-clock (ATC) reach steady state—the point when the amount of opioid excreted between doses approximates the amount added by subsequent doses—in 4 to 5 half-lives; it takes about 24 hours for morphine, oxycodone, hydrocodone, or IV fentanyl but several days for methadone, levorphanol, or TD opioids (Pasero & McCaffery, 2011; Portenoy & Ahmed, 2014). Scheduled baseline doses should be titrated upward *only* after steady state is reached, but prn doses of a short-acting opioid—10% to 15% of the current 24-hour opioid dose—should always be permitted for uncontrolled pain. 

Baseline scheduled plus prn doses per 24 hours, current pain intensity, and any adverse effects are considered in dose increases (or decreases). Sustained release and TD opioids, which are best for patients with satisfactory analgesia, decrease serum opioid fluctuations, extend analgesia, draw patients’ attention away from pain and pill-taking, allow greater focus on "normal" aspects of life, and may enhance adherence (Rauck, 2009). Conversely, opioids taken for longer than 2 weeks cannot be abruptly stopped, even if pain is suddenly relieved (e.g., after a neuroablative procedure) because of physical dependence. A slow, gradual dose taper by 25% to 50% every 2 to 4 days, while monitoring carefully for signs and symptoms of withdrawal, is recommended.

**Transdermal Opioids**

Transdermal fentanyl (TDF) or transdermal buprenorphine (TDB) may be an option for opioid-tolerant patients with no po intake, to minimize pill-taking behaviors or to enhance adherence. Transdermal patches are formulated as a drug-containing reservoir (fentanyl) with a rate-controlling drug delivery membrane or as an adhesive polymer layer with a drug-incorporated matrix (fentanyl or buprenorphine) in which the drug amount controls delivery. Impaired skin integrity or heat (fever or a heating pad over a patch) can increase drug delivery with either TD system (Moore, Sathyan, Richarz, Natarajan, & Vandenbossche, 2012; Prodduturi et al., 2010). Magnetic resonance imaging (MRI) may cause a burn beneath patches within a scanned area, and so patches should be removed before and reapplied after an MRI (Portenoy & Ahmed, 2014). 

After first-patch application, a TD opioid slowly diffuses through skin layers, forming a "skin depot," and then absorbs into capillaries. Serum levels are measurable in 8 to 12 hours; TDF levels off at 24 to 36 hours, and TDB levels off at 36 to 48 hours (Andresen et al, 2010; Khanna & Pillarisetti, 2015; Kornick et al., 2001). As-needed opioid doses by another route are necessary, especially until steady state. The skin depot effect is also important for 24 hours after TDF and for at least 30 hours after TDB is discontinued. Adverse effects do not resolve until TD opioid serum level falls to less than 50%.

**Figure 3:**
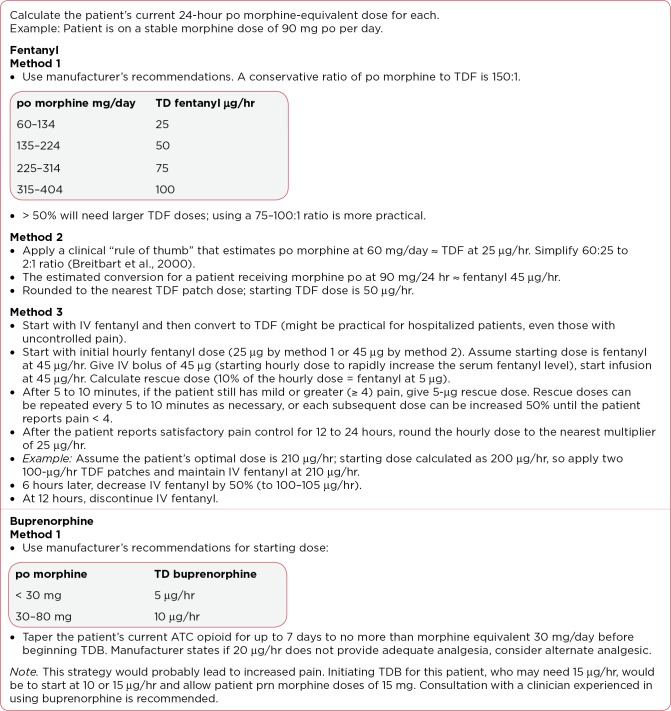
Converting to a transdermal opioid. po = oral; TDF = transdermal fentanyl; IV = intravenous; ATC = around-the-clock; TDB = transdermal buprenorphine; prn = as needed. Information from Janssen (2017); Kornick et al. (2001); Nomura et al. (2011); Purdue Pharma (2017).

Ways to switch to TD opioids, illustrated in Figure 3, include manufacturers’ recommendations (Janssen, 2017; Purdue Pharma, 2017). With fentanyl, some clinicians use a "rule of thumb" or start IV fentanyl and then convert to TDF (Breitbart et al., 2000; Kornick et al., 2001; Nomura et al., 2011). There are fewer reports of TDB for cancer-related pain.

In one study, 42 patients with chronic or cancer pain had poor analgesia or intolerable side effects with po opioids (morphine equivalent > 120 mg/day) were switched to TDB ≥ 52.5 mg/hour (Freye, Anderson-Hillemacher, Ritzdorf, & Levy, 2007). The proportion of patients who reported good/very good pain relief increased from 5% before to 76% after switching (*p* < .001).

A larger prospective multicenter study of 520 cancer patients with moderate to severe pain were randomly assigned to morphine SR, oxycodone SR, TDF, or TDB and followed for 28 days (Corli et al., 2016). Pain decreases, numbers of responders, and safety profiles were similar among these opioids, leading the authors to conclude any one could be recommended as a first-line option for moderate to severe cancer pain.

Fine and Portenoy (2009) addressed research-based manufacturers’ recommendations for conversion from another opioid to TDF, which incorporated a safety factor. This conservative recommendation does not require a reduction in the calculated equianalgesic dose. Similarly, some patients have been switched to TDB using a 75:1 or lower ratio (Freye et al., 2007).

**Transmucosal Fentanyl**

Transitory, breakthrough cancer pain (BTCP) episodes usually peak within a few minutes and last for about 30 to 35 minutes (Hagen, Biondo, & Stiles, 2008). Oral morphine or oxycodone IR may be ordered, but they have low bioavailability (30%–50%) and do not provide meaningful pain relief for 30 to 45 minutes. On the other hand, TM fentanyl (TMF) products have more rapid onset, peak, and fall off.

One prospective study compared po morphine and sublingual TMF for BTCP in cancer patients (Rivera, Garrido, Velasco, de Enciso, & Clavarana, 2014). Transmucosal fentanyl was superior to morphine in providing pain relief at 4 measurement times over 30 days, with significantly shorter times to effective doses (6.6 days vs. 13.3 days) and more dissatisfaction with morphine. 

Clinicians must review TMF products to determine which might be best for their patients. Several oral (e.g., sublingual fentanyl citrate, sublingual tablets, sublingual spray, buccal soluble film, and buccal tablets) and intranasal TMF products (aqueous or pectin sprays) are similar but not exactly the same. For instance, sublingual TMF absorbs more rapidly than buccal because of differences in mucosal thickness, and 50% to 70% of some oral TMF products dissolve in saliva and are swallowed. The bioavailability of TMF products varies from as low as 30% to 89% with aqueous intranasal fentanyl spray because of high nasal mucosal vascularity and permeability (Corli & Roberto, 2014). Time to maximal pain relief is 15 to 30 minutes with po and about 10 minutes with intranasal TMF (Zeppetella, Davies, Eijgelshoven, & Jansen, 2014).

Manufacturers incorporate bioavailability data into their formulated doses, and different products are not directly interchangeable. No matter the baseline dose, experts recommend using the lowest TDF dose (any selected product) when starting TMF for breakthrough pain (Fine & Portenoy, 2009). A first TMF dose that does not relieve pain within 15 minutes can be repeated once, and patients who use more than 4 doses a day may need a higher baseline opioid dose (Minkowitz, Bull, Brownlow, Parikh, & Rauck, 2016).

## OPIOID ROTATION 

Possible reasons for switching a patient from one opioid to a different opioid include inadequate analgesia despite aggressive dose titration, dose-limiting adverse effects, clinical status change, serious drug-drug interactions, financial or drug-availability issues, or hyperalgesia (Fine & Portenoy, 2009). Clinicians use equianalgesia tables or algorithms for decisions about opioid rotation (Table 6), which are, at best, "ballpark" estimates of approximately equivalent doses. They are based on extrapolation from studies not designed to evaluate equianalgesia, and data are limited by wide confidence intervals and large standard deviations (Mercadante & Caraceni, 2011). 

**Table 6:**
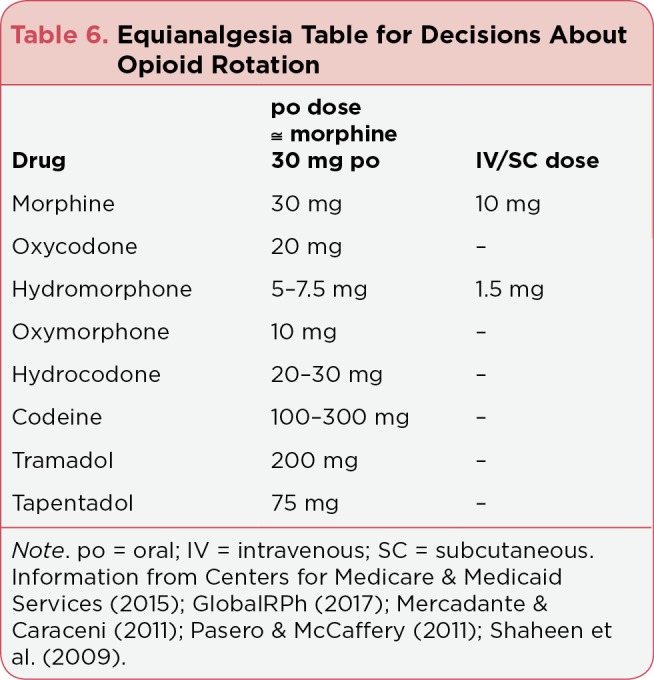
Equianalgesia Table for Decisions About Opioid Rotation

An ad hoc pain expert panel formulated guidelines for calculating doses of new opioids (Fine & Portenoy, 2009). Step one recommends to always incorporate an automatic safety factor: a 25% to 50% reduction in a calculated equianalgesic dose to account for incomplete cross-tolerance among opioids (Prommer, 2015; Ripamonti et al., 2012; Shaheen, Walsh, Lasheen, Davis, & Lagman, 2009). Inherent drug differences at μ receptors means 50% to 90% of patients who switched to a different opioid experience better pain control and fewer adverse effects (Fine & Portenoy, 2009; Mercadante & Bruera, 2016; Portenoy & Ahmed, 2014). Patients need close monitoring for prn doses and perhaps rapid baseline dose escalation. If a patient is switching because of hyperalgesia, the patient’s opioid dose before the onset of hyperalgesia should be used for the conversion ratio (Yi & Pryzbylkowski, 2015). 

The second recommended step is to consider any patient or drug characteristics that support further equianalgesic dose adjustment (Fine & Portenoy, 2009). For example, age, comorbidities, or treatment-related factors might be important, as well as direct or indirect drug issues, such as drug sensitivities, adherence, financial issues or third-party payment, or safety concerns. In particular, the relationship between methadone dose and potency is linear; when switching from a high dose of another opioid to methadone, the calculated methadone should be reduced by 75% to 90% (Mercadante & Caraceni, 2011; Shaheen et al., 2009). Conversely, reductions can be smaller with lower opioid doses. The mathematical calculation of equianalgesic doses is simple, but prescribers should collaborate with another professional colleague so each one independently calculates equianalgesic doses, double-checks calculations, and discusses incorporating safety factors to their rationale for new opioid dosing—all of which increase clinical skills and patient safety.

## CONCLUSIONS

Opioid analgesics are one cornerstone of cancer pain management, the others being nonopioid analgesics, thorough baseline and timely reassessments, and interprofessional, patient-centered collaborative management that includes nondrug measures and interventions. Collaborative management, particularly calling upon the expertise of oncology pharmacists and the rich clinical experiences of other colleagues in fields such as palliative or supportive care, pain centers, physical therapy, and complemental therapies can enhance patient safety and quality of life, even for those with moderate to severe pain at any point along the cancer trajectory. In terms of opioids, this is especially relevant when using more unfamiliar drugs or prescribing higher doses than most patients require. Continuing education, clinical and didactic, will also help advanced practitioners make substantial and meaningful contributions to optimal pain management for individuals with cancer.

## References

[1] Anchersen Katinka, Clausen Thomas, Gossop Michael, Hansteen Viggo, Waal Helge (2009). Prevalence and clinical relevance of corrected QT interval prolongation during methadone and buprenorphine treatment: a mortality assessment study.. *Addiction (Abingdon, England)*.

[2] Andresen Trine, Upton Richard N, Foster David J R, Christrup Lona L, Arendt-Nielsen Lars, Drewes Asbjørn M (2011). Pharmacokinetic/pharmacodynamic relationships of transdermal buprenorphine and fentanyl in experimental human pain models.. *Basic & clinical pharmacology & toxicology*.

[3] Barakat Neveen H, Atayee Rabia S, Best Brookie M, Pesce Amadeo J (2012). Relationship between the concentration of hydrocodone and its conversion to hydromorphone in chronic pain patients using urinary excretion data.. *Journal of analytical toxicology*.

[4] Barclay Joshua S, Owens Justine E, Blackhall Leslie J (2014). Screening for substance abuse risk in cancer patients using the Opioid Risk Tool and urine drug screen.. *Supportive care in cancer : official journal of the Multinational Association of Supportive Care in Cancer*.

[5] Beakley B D, Kaye A M, Kaye A D (2015). Tramadol, pharmacology, side effects, and serotonin syndrome: A review.. *Pain Physician*.

[6] Berling I, Whyte I M, Isbister G K (2013). Oxycodone overdose causes naloxone responsive coma and QT prolongation.. *QJM : monthly journal of the Association of Physicians*.

[7] Bosilkovska Marija, Walder Bernhard, Besson Marie, Daali Youssef, Desmeules Jules (2012). Analgesics in patients with hepatic impairment: pharmacology and clinical implications.. *Drugs*.

[8] Branford R, Droney J, Ross J R (2012). Opioid genetics: the key to personalized pain control?. *Clinical genetics*.

[9] Brant Jeannine M (2010). Practical approaches to pharmacologic management of pain in older adults with cancer.. *Oncology nursing forum*.

[10] Breitbart W, Chandler S, Eagel B, Ellison N, Enck R E, Lefkowitz M, Payne R (2000). An alternative algorithm for dosing transdermal fentanyl for cancer-related pain.. *Oncology*.

[11] Brennan Michael J (2013). The effect of opioid therapy on endocrine function.. *The American journal of medicine*.

[12] Brock Christina, Olesen Søren Schou, Olesen Anne Estrup, Frøkjaer Jens Brøndum, Andresen Trine, Drewes Asbjørn Mohr (2012). Opioid-induced bowel dysfunction: pathophysiology and management.. *Drugs*.

[13] Bush Shirley H, Bruera Eduardo (2009). The assessment and management of delirium in cancer patients.. *The oncologist*.

[14] Buss Tomasz, Leppert Wojciech (2014). Opioid-induced endocrinopathy in cancer patients: an underestimated clinical problem.. *Advances in therapy*.

[15] Caraceni Augusto, Hanks Geoffrey, Kaasa Stein, Bennett Michael I, Brunelli Cinzia, Cherny Nathan, Dale Ola, De Conno Franco, Fallon Marie, Hanna Magdi, Haugen Dagny Faksvåg, Juhl Gitte, King Samuel, Klepstad Pål, Laugsand Eivor A, Maltoni Marco, Mercadante Sebastiano, Nabal Maria, Pigni Alessandra, Radbruch Lukas, Reid Colette, Sjogren Per, Stone Patrick C, Tassinari Davide, Zeppetella Giovambattista (2012). Use of opioid analgesics in the treatment of cancer pain: evidence-based recommendations from the EAPC.. *The Lancet. Oncology*.

[16] Centers for Medicare & Medicaid Services. (2015). Opioid morphine EQ conversion factors.. https://www.cms.gov/Medicare/Prescription-Drug-Coverage/PrescriptionDrugCovContra/Downloads/Opioid-Morphine-EQ-Conversion-Factors-March-2015.pdf.

[17] Cherny N, Ripamonti C, Pereira J, Davis C, Fallon M, McQuay H, Mercadante S, Pasternak G, Ventafridda V (2001). Strategies to manage the adverse effects of oral morphine: an evidence-based report.. *Journal of clinical oncology : official journal of the American Society of Clinical Oncology*.

[18] Chou Roger, Cruciani Ricardo A, Fiellin David A, Compton Peggy, Farrar John T, Haigney Mark C, Inturrisi Charles, Knight John R, Otis-Green Shirley, Marcus Steven M, Mehta Davendra, Meyer Marjorie C, Portenoy Russell, Savage Seddon, Strain Eric, Walsh Sharon, Zeltzer Lonnie (2014). Methadone safety: a clinical practice guideline from the American Pain Society and College on Problems of Drug Dependence, in collaboration with the Heart Rhythm Society.. *The journal of pain : official journal of the American Pain Society*.

[19] Chowdhury M M, Board R (2009). Morphine-induced hallucinations—Resolution with switching to oxycodone: A case report and review of the literature.. *Cases Journal*.

[20] Clemens Katri E, Faust Markus, Jaspers Birgit, Mikus Gerd (2013). Pharmacological treatment of constipation in palliative care.. *Current opinion in supportive and palliative care*.

[21] Colameco Stephen, Coren Joshua S (2009). Opioid-induced endocrinopathy.. *The Journal of the American Osteopathic Association*.

[22] Collins S L, Faura C C, Moore R A, McQuay H J (1998). Peak plasma concentrations after oral morphine: a systematic review.. *Journal of pain and symptom management*.

[23] Corli O, Floriani I, Roberto A, Montanari M, Galli F, Greco M T, Caraceni A, Kaasa S, Dragani T A, Azzarello G, Luzzani M, Cavanna L, Bandieri E, Gamucci T, Lipari G, Di Gregorio R, Valenti D, Reale C, Pavesi L, Iorno V, Crispino C, Pacchioni M, Apolone G (2016). Are strong opioids equally effective and safe in the treatment of chronic cancer pain? A multicenter randomized phase IV 'real life' trial on the variability of response to opioids.. *Annals of oncology : official journal of the European Society for Medical Oncology*.

[24] Corli O, Roberto A (2014). Pharmacological and clinical differences among transmucosal fentanyl formulations for the treatment of breakthrough cancer pain: a review article.. *Minerva anestesiologica*.

[25] CredibleMeds. ((2017). CredibleMeds: Home page.. https://www.crediblemeds.org/.

[26] Crews K R, Gaedigk A, Dunnenberger H M, Leeder J S, Klein T E, Caudle K E, Haidar C E, Shen D D, Callaghan J T, Sadhasivam S, Prows C A, Kharasch E D, Skaar T C (2014). Clinical Pharmacogenetics Implementation Consortium guidelines for cytochrome P450 2D6 genotype and codeine therapy: 2014 update.. *Clinical pharmacology and therapeutics*.

[27] Dahan Albert, Aarts Leon, Smith Terry W (2010). Incidence, Reversal, and Prevention of Opioid-induced Respiratory Depression.. *Anesthesiology*.

[28] Darpo Borje, Zhou Meijian, Bai Stephen A, Ferber Georg, Xiang Qinfang, Finn Andrew (2016). Differentiating the Effect of an Opioid Agonist on Cardiac Repolarization From µ-Receptor-mediated, Indirect Effects on the QT Interval: A Randomized, 3-way Crossover Study in Healthy Subjects.. *Clinical therapeutics*.

[29] Davis M P (2012). Twelve reasons for considering buprenorphine as a frontline analgesic in the management of pain.. *Journal of Supportive Oncology,*.

[30] Davis M P, Walsh D (2001). Methadone for relief of cancer pain: a review of pharmacokinetics, pharmacodynamics, drug interactions and protocols of administration.. *Supportive care in cancer : official journal of the Multinational Association of Supportive Care in Cancer*.

[31] De Gregori Manuela, Diatchenko Luda, Ingelmo Pablo M, Napolioni Valerio, Klepstad Pal, Belfer Inna, Molinaro Valeria, Garbin Giulia, Ranzani Guglielmina N, Alberio Giovanni, Normanno Marco, Lovisari Federica, Somaini Marta, Govoni Stefano, Mura Elisa, Bugada Dario, Niebel Thekla, Zorzetto Michele, De Gregori Simona, Molinaro Mariadelfina, Fanelli Guido, Allegri Massimo (2016). Human Genetic Variability Contributes to Postoperative Morphine Consumption.. *The journal of pain : official journal of the American Pain Society*.

[32] De Maddalena C, Bellini M, Berra M, Meriggiola C, Aloisi A M (2012). Opioid-induced hypogonadism: Why and how to treat it.. *Pain Physician*.

[33] DePriest A Z, Puet B L, Holt A C, Roberts A, Cone E J (2015). Metabolism and Disposition of Prescription Opioids: A Review.. *Forensic science review*.

[34] Donnelly Sinead, Davis Mellar P, Walsh Declan, Naughton Michael (2002). Morphine in cancer pain management: a practical guide.. *Supportive care in cancer : official journal of the Multinational Association of Supportive Care in Cancer*.

[35] Enggaard Thomas P, Poulsen Lars, Arendt-Nielsen Lars, Brøsen Kim, Ossig Joachim, Sindrup Søren H (2006). The analgesic effect of tramadol after intravenous injection in healthy volunteers in relation to CYP2D6.. *Anesthesia and analgesia*.

[36] Fine Perry G, Portenoy Russell K (2009). Establishing "best practices" for opioid rotation: conclusions of an expert panel.. *Journal of pain and symptom management*.

[37] Freye Enno, Anderson-Hillemacher Astrid, Ritzdorf Ingrid, Levy Joseph Victor (2007). Opioid rotation from high-dose morphine to transdermal buprenorphine (Transtec) in chronic pain patients.. *Pain practice : the official journal of World Institute of Pain*.

[38] Fukshansky M, Are M, Burton A W (2005). The role of opioids in cancer pain management.. *Pain Practice*.

[39] GlobalRPh. (2017). vanced opioid converter.. http://www.globalrph.com/opioidconverter2.htm.

[40] Guay David R P (2009). Is tapentadol an advance on tramadol?. *The Consultant pharmacist : the journal of the American Society of Consultant Pharmacists*.

[41] Gudin Jeffrey (2012). Opioid therapies and cytochrome p450 interactions.. *Journal of pain and symptom management*.

[42] Gudin Jeffrey, Fudin Jeffrey, Nalamachu Srinivas (2016). Levorphanol use: past, present and future.. *Postgraduate medicine*.

[43] Hagen Neil A, Biondo Patricia, Stiles Carla (2008). Assessment and management of breakthrough pain in cancer patients: current approaches and emerging research.. *Current pain and headache reports*.

[44] Hoskin P J, Hanks G W, Aherne G W, Chapman D, Littleton P, Filshie J (1989). The bioavailability and pharmacokinetics of morphine after intravenous, oral and buccal administration in healthy volunteers.. *British journal of clinical pharmacology*.

[45] Houlihan David J (2004). Serotonin syndrome resulting from coadministration of tramadol, venlafaxine, and mirtazapine.. *The Annals of pharmacotherapy*.

[46] Ingelman-Sundberg M (n.d.). The human cytochrome P450 (CYP) allele nomenclature database.. http://www.cypalleles.ki.se.

[47] Isbister Geoffrey K (2015). Risk assessment of drug-induced QT prolongation.. *Australian prescriber*.

[48] Janssen. (2017). Duragesic (fentanyl transdermal system) package insert.. http://www.duragesic.com/assets/pdf/duragesic_0.pdf.

[49] Kalso Eija (2005). Oxycodone.. *Journal of pain and symptom management*.

[50] Kao David P, Haigney Mark C P, Mehler Philip S, Krantz Mori J (2015). Arrhythmia associated with buprenorphine and methadone reported to the Food and Drug Administration.. *Addiction (Abingdon, England)*.

[51] Katz Nathaniel, Mazer Norman A (2009). The impact of opioids on the endocrine system.. *The Clinical journal of pain*.

[52] Keller Guillermo Alberto, Ponte Marcelo L, Di Girolamo Guillermo (2010). Other drugs acting on nervous system associated with QT-interval prolongation.. *Current drug safety*.

[53] Keller Guillermo A, Etchegoyen María C V, Fernandez Nicolás, Olivera Nancy M, Quiroga Patricia N, Belloso Waldo H, Diez Roberto A, Di Girolamo Guillermo (2016). Tramadol Induced QTc-Interval Prolongation: Prevalence, Clinical Factors and Correlation to Plasma Concentrations.. *Current drug safety*.

[54] Khanna Ish K, Pillarisetti Sivaram (2015). Buprenorphine - an attractive opioid with underutilized potential in treatment of chronic pain.. *Journal of pain research*.

[55] King Samuel James, Reid Colette, Forbes Karen, Hanks Geoffrey (2011). A systematic review of oxycodone in the management of cancer pain.. *Palliative medicine*.

[56] Kirchheiner J, Schmidt H, Tzvetkov M, Keulen J-T H A, Lötsch J, Roots I, Brockmöller J (2007). Pharmacokinetics of codeine and its metabolite morphine in ultra-rapid metabolizers due to CYP2D6 duplication.. *The pharmacogenomics journal*.

[57] Klepstad Pål, Kaasa Stein, Borchgrevink Petter C (2011). Starting step III opioids for moderate to severe pain in cancer patients: dose titration: a systematic review.. *Palliative medicine*.

[58] Kornick C A, Santiago-Palma J, Khojainova N, Primavera L H, Payne R, Manfredi P L (2001). A safe and effective method for converting cancer patients from intravenous to transdermal fentanyl.. *Cancer*.

[59] Krantz Mori J, Martin Judith, Stimmel Barry, Mehta Davendra, Haigney Mark C P (2009). QTc interval screening in methadone treatment.. *Annals of internal medicine*.

[60] Kumar Maansi G, Lin Senshang (2007). Hydromorphone in the management of cancer-related pain: an update on routes of administration and dosage forms.. *Journal of pharmacy & pharmaceutical sciences : a publication of the Canadian Society for Pharmaceutical Sciences, Societe canadienne des sciences pharmaceutiques*.

[61] Lam Lisa H, Pirrello Rosene D, Ma Joseph D (2016). A Case-Based Approach to Integrating Opioid Pharmacokinetic and Pharmacodynamic Concepts in Cancer Pain Management.. *Journal of clinical pharmacology*.

[62] Leppert W (2009). Tramadol as an analgesic for mild to moderate cancer pain.. *Pharmacological Reports*.

[63] Leppert Wojciech (2011). CYP2D6 in the metabolism of opioids for mild to moderate pain.. *Pharmacology*.

[64] Loitman J E (2011). Fast facts and concepts. Levorphanol #240.. *Journal of Palliative Medicine*.

[65] Manini A F, Stimmel B, Vlahov D (2014). Racial susceptibility for QT prolongation in acute drug overdoses.. *Journal of Electrocardiology*.

[66] Marinangeli Franco, Ciccozzi Alessandra, Leonardis Marco, Aloisio Luca, Mazzei Anna, Paladini Antonella, Porzio Giampiero, Marchetti Paolo, Varrassi Giustino (2004). Use of strong opioids in advanced cancer pain: a randomized trial.. *Journal of pain and symptom management*.

[67] Mayyas Fadia, Fayers Peter, Kaasa Stein, Dale Ola (2010). A systematic review of oxymorphone in the management of chronic pain.. *Journal of pain and symptom management*.

[68] McNulty Jack P (2007). Can levorphanol be used like methadone for intractable refractory pain?. *Journal of palliative medicine*.

[69] Mercadante Sebastiano (2015). Opioid metabolism and clinical aspects.. *European journal of pharmacology*.

[70] Mercadante S (2016). Opioid-related adverse effects.. *Xiangya Medicine*.

[71] Mercadante Sebastiano, Bruera Eduardo (2016). Opioid switching in cancer pain: From the beginning to nowadays.. *Critical reviews in oncology/hematology*.

[72] Mercadante Sebastiano, Caraceni Augusto (2011). Conversion ratios for opioid switching in the treatment of cancer pain: a systematic review.. *Palliative medicine*.

[73] Minkowitz Harold, Bull Janet, Brownlow R Charles, Parikh Neha, Rauck Richard (2016). Long-term safety of fentanyl sublingual spray in opioid-tolerant patients with breakthrough cancer pain.. *Supportive care in cancer : official journal of the Multinational Association of Supportive Care in Cancer*.

[74] Moore Kenneth T, Sathyan Gayatri, Richarz Ute, Natarajan Jaya, Vandenbossche Joris (2012). Randomized 5-treatment crossover study to assess the effects of external heat on serum fentanyl concentrations during treatment with transdermal fentanyl systems.. *Journal of clinical pharmacology*.

[75] Murray Alison, Hagen Neil A (2005). Hydromorphone.. *Journal of pain and symptom management*.

[76] Nafziger Anne N, Bertino Joseph S (2009). Utility and application of urine drug testing in chronic pain management with opioids.. *The Clinical journal of pain*.

[77] Nalamachu Srinivas R (2012). Opioid rotation in clinical practice.. *Advances in therapy*.

[78] National Association of Boards of Pharmacy. (2017). PMP InterConnect.. https://nabp.pharmacy/initiatives/pmp-interconnect/.

[79] Nomura Motoo, Kamata Minoru, Kojima Hiroyuki, Hayashi Kenji, Kozai Masasuke, Sawada Satoshi (2011). Six- versus 12-h conversion method from intravenous to transdermal fentanyl in chronic cancer pain: a randomized study.. *Supportive care in cancer : official journal of the Multinational Association of Supportive Care in Cancer*.

[80] Olkkola Klaus T, Kontinen Vesa K, Saari Teijo I, Kalso Eija A (2013). Does the pharmacology of oxycodone justify its increasing use as an analgesic?. *Trends in pharmacological sciences*.

[81] Orliaguet Gilles, Hamza Jamil, Couloigner Vincent, Denoyelle Françoise, Loriot Marie-Anne, Broly Franck, Garabedian Erea Noel (2015). A case of respiratory depression in a child with ultrarapid CYP2D6 metabolism after tramadol.. *Pediatrics*.

[82] Pasero C, McCaffery M (2011). *Pain assessment and pharmacologic management*.

[83] Pasternak Gavril W (2014). Opioids and their receptors: Are we there yet?. *Neuropharmacology*.

[84] Pathan Hasan, Williams John (2012). Basic opioid pharmacology: an update.. *British journal of pain*.

[85] Pergolizzi J, Aloisi A M, Dahan A, Filitz J, Langford R, Likar R, Weinbroum A A (2010). Current knowledge of buprenorphine and its unique pharmacological profile.. *Pain Practice*.

[86] Pham Thien C, Fudin Jeffrey, Raffa Robert B (2015). Is levorphanol a better option than methadone?. *Pain medicine (Malden, Mass.)*.

[87] PharmGKB. (2017). Annotation of US Food and Drug Administration (FDA) label information for codeine and CYP2D6.. https://www.pharmgkb.org/label/PA166104916.

[88] Pigni Alessandra, Brunelli Cinzia, Caraceni Augusto (2011). The role of hydromorphone in cancer pain treatment: a systematic review.. *Palliative medicine*.

[89] Porreca F, Ossipov M H (2009). Nausea and vomiting side effects with opioid analgesics during treatment of chronic pain: Mechanisms, implications, and management options.. *Pain Medicine*.

[90] Portenoy Russell K, Ahmed Ebtesam (2014). Principles of opioid use in cancer pain.. *Journal of clinical oncology : official journal of the American Society of Clinical Oncology*.

[91] Pöyhiä R, Seppälä T, Olkkola K T, Kalso E (1992). The pharmacokinetics and metabolism of oxycodone after intramuscular and oral administration to healthy subjects.. *British journal of clinical pharmacology*.

[92] Prescrire Editorial Staff (2015). ’Weak’ opioid analgesics: Codeine, dihydrocodeine, and tramadol: No less risky than morphine.. *La Revue Prescrire*.

[93] Prodduturi Suneela, Sadrieh Nakissa, Wokovich Anna M, Doub William H, Westenberger Benjamin J, Buhse Lucinda (2010). Transdermal delivery of fentanyl from matrix and reservoir systems: effect of heat and compromised skin.. *Journal of pharmaceutical sciences*.

[94] Prommer Eric (2006). Oxymorphone: a review.. *Supportive care in cancer : official journal of the Multinational Association of Supportive Care in Cancer*.

[95] Prommer Eric (2012). Methylphenidate: established and expanding roles in symptom management.. *The American journal of hospice & palliative care*.

[96] Prommer Eric (2014). Levorphanol: revisiting an underutilized analgesic.. *Palliative care*.

[97] Prommer Eric E (2015). Pharmacological Management of Cancer-Related Pain.. *Cancer control : journal of the Moffitt Cancer Center*.

[98] Purdue Pharma. (2017). Butrans (buprenorphine) transdermal system package insert.. http://app.purduepharma.com/xmlpublishing/pi.aspx?id=b.

[99] Rauck R L (2009). What is the case for prescribing long-acting opioids over short-acting opioids for patients with chronic pain? A critical review.. *Pain Practice*.

[100] Rauck Richard L (2013). Treatment of opioid-induced constipation: focus on the peripheral μ-opioid receptor antagonist methylnaltrexone.. *Drugs*.

[101] Reichert C, Reichert P, Monnet-Tschudi F, Kupferschmidt H, Ceschi A, Rauber-Lüthy C (2014). Seizures after single-agent overdose with pharmaceutical drugs: analysis of cases reported to a poison center.. *Clinical toxicology (Philadelphia, Pa.)*.

[102] Ripamonti C I, Santini D, Maranzano E, Berti M, Roila F (2012). Management of cancer pain: ESMO clinical practice guidelines.. *Annals of Oncology*.

[103] Velázquez Rivera Ignacio, Muñoz Garrido José Carlos, García Velasco Pilar, España Ximénez de Enciso Inmaculada, Velázquez Clavarana Lourdes (2014). Efficacy of sublingual fentanyl vs. oral morphine for cancer-related breakthrough pain.. *Advances in therapy*.

[104] Shaheen Philip E, Walsh Declan, Lasheen Wael, Davis Mellar P, Lagman Ruth L (2009). Opioid equianalgesic tables: are they all equally dangerous?. *Journal of pain and symptom management*.

[105] Sivanesan Eellan, Gitlin Melvin C, Candiotti Keith A (2016). Opioid-induced Hallucinations: A Review of the Literature, Pathophysiology, Diagnosis, and Treatment.. *Anesthesia and analgesia*.

[106] Smith H S (2009). Clinical pharmacology of oxymorphone.. *Pain Medicine*.

[107] Smith H S (2011). The metabolism of opioid agents and the clinical impact of their active metabolites.. *Clinical Journal of Pain*.

[108] Stamer Ulrike M, Stüber Frank, Muders Thomas, Musshoff Frank (2008). Respiratory depression with tramadol in a patient with renal impairment and CYP2D6 gene duplication.. *Anesthesia and analgesia*.

[109] Stringer John, Welsh Christopher, Tommasello Anthony (2009). Methadone-associated Q-T interval prolongation and torsades de pointes.. *American journal of health-system pharmacy : AJHP : official journal of the American Society of Health-System Pharmacists*.

[110] Stuart-Harris R, Joel S P, McDonald P, Currow D, Slevin M L (2000). The pharmacokinetics of morphine and morphine glucuronide metabolites after subcutaneous bolus injection and subcutaneous infusion of morphine.. *British journal of clinical pharmacology*.

[111] Tassinari Davide, Drudi Fabrizio, Rosati Marta, Tombesi Paola, Sartori Sergio, Maltoni Marco (2011). The second step of the analgesic ladder and oral tramadol in the treatment of mild to moderate cancer pain: a systematic review.. *Palliative medicine*.

[112] Tremblay Johanne, Hamet Pavel (2010). Genetics of pain, opioids, and opioid responsiveness.. *Metabolism: clinical and experimental*.

[113] Trescot A M, Faynboym S (2014). A review of the role of genetic testing in pain medicine.. *Pain Physician*.

[114] Tsutaoka Ben T, Ho Raymond Y, Fung Stacey M, Kearney Thomas E (2015). Comparative Toxicity of Tapentadol and Tramadol Utilizing Data Reported to the National Poison Data System.. *The Annals of pharmacotherapy*.

[115] US Food and Drug Administration. (2017). Opioid pain medicines: Drug safety communication - new safety warnings added to prescription opioid medications.. https://www.fda.gov/Safety/MedWatch/SafetyInformation/SafetyAlertsforHumanMedicalProducts/ucm491715.htm.

[116] Vallejo R, Barkin R L, Wang V C (2011). Pharmacology of opioids in the treatment of chronic pain syndromes.. *Pain Physician*.

[117] Vandael Eline, Vandenberk Bert, Vandenberghe Joris, Willems Rik, Foulon Veerle (2017). Risk factors for QTc-prolongation: systematic review of the evidence.. *International journal of clinical pharmacy*.

[118] Veal Felicity C, Peterson Gregory M (2015). Pain in the Frail or Elderly Patient: Does Tapentadol Have a Role?. *Drugs & aging*.

[119] Vella-Brincat Jane, Macleod A D (2007). Adverse effects of opioids on the central nervous systems of palliative care patients.. *Journal of pain & palliative care pharmacotherapy*.

[120] Virk Michael S, Arttamangkul Seksiri, Birdsong William T, Williams John T (2009). Buprenorphine is a weak partial agonist that inhibits opioid receptor desensitization.. *The Journal of neuroscience : the official journal of the Society for Neuroscience*.

[121] Wang Yun, Sands Laura P, Vaurio Linnea, Mullen E Ann, Leung Jacqueline M (2007). The effects of postoperative pain and its management on postoperative cognitive dysfunction.. *The American journal of geriatric psychiatry : official journal of the American Association for Geriatric Psychiatry*.

[122] Webster Lynn, Andrews Megan, Stoddard Gregory (2003). Modafinil treatment of opioid-induced sedation.. *Pain medicine (Malden, Mass.)*.

[123] Wei Leslie A, Fearing Michael A, Sternberg Eliezer J, Inouye Sharon K (2008). The Confusion Assessment Method: a systematic review of current usage.. *Journal of the American Geriatrics Society*.

[124] Werneke U, Jamshidi F, Taylor D M, Ott M (2016). Conundrums in neurology: Diagnosing serotonin syndrome – a meta-analysis of cases.. *BMC Neurology*.

[125] Wickham Rita J (2017). Managing Constipation in Adults With Cancer.. *Journal of the advanced practitioner in oncology*.

[126] Wiffen P J, Derry S, Naessens K, Bell R F (2015). Oral tapentadol for cancer pain.. *Cochrane Database of Systematic Reviews*.

[127] Wirz S, Wartenberg H C, Nadstawek J (2008). Less nausea, emesis, and constipation comparing hydromorphone and morphine? A prospective open-labeled investigation on cancer pain.. *Supportive care in cancer : official journal of the Multinational Association of Supportive Care in Cancer*.

[128] Yi Peter, Pryzbylkowski Peter (2015). Opioid Induced Hyperalgesia.. *Pain medicine (Malden, Mass.)*.

[129] Youssef F, Pater A, Shehata M (2015). Opioid-induced hyperalgesia.. *Pain & Relief*.

[130] Zeppetella G, Davies A, Eijgelshoven I, Jansen J P (2014). A network meta-analysis of the efficacy of opioid analgesics for the management of breakthrough cancer pain episodes.. *Journal of Pain and Symptom Management*.

